# Supplementary material for: A Comprehensive Assessment of Ultraviolet-Radiation-Induced Mutations in Flammulina filiformis Using Whole-Genome Resequencing
Source: J Fungi (Basel). 2024 Mar 20;10(3):228. doi: 10.3390/jof10030228 (PMC10971301; doi:10.3390/jof10030228)
Supplement: Supplementary file 1 [file jof-10-00228-s001.zip › Supplementary Material S8/KEGG annotation/out/64381550635650.os/KO/out_map/map04620.html]

KEGG PATHWAY: Toll-like receptor signaling pathway - Reference pathway


|  |  |
| --- | --- |
| **Toll-like receptor signaling pathway - Reference pathway** |  |

[
Pathway menu
| Organism menu
| Pathway entry
| Show description
| User data mapping
]

Specific families of pattern recognition receptors are responsible for detecting microbial pathogens and generating innate immune responses. Toll-like receptors (TLRs) are membrane-bound receptors identified as homologs of Toll in Drosophila. Mammalian TLRs are expressed on innate immune cells, such as macrophages and dendritic cells, and respond to the membrane components of Gram-positive or Gram-negative bacteria. Pathogen recognition by TLRs provokes rapid activation of innate immunity by inducing production of proinflammatory cytokines and upregulation of costimulatory molecules. TLR signaling pathways are separated into two groups: a MyD88-dependent pathway that leads to the production of proinflammatory cytokines with quick activation of NF-{kappa}B and MAPK, and a MyD88-independent pathway associated with the induction of IFN-beta and IFN-inducible genes, and maturation of dendritic cells with slow activation of NF-{kappa}B and MAPK.

|  |  |
| --- | --- |
| Reference pathway | 100% |
